# Community-Based Health Planning and Services (CHPS) concept and access to healthcare delivery in Sefwi Wiawso Municipal, Ghana

**DOI:** 10.1186/s12913-024-11179-6

**Published:** 2024-06-17

**Authors:** Abraham D. Koyaara, Benjamin Noble Adjei, Eric Adjei Boadu, Edward T. Dassah

**Affiliations:** 1 Municipal Health Directorate-Ghana Health Service, Sefwi Wiawso, Western North, Ghana; 2 Department of Epidemiology and Biostatistics, Kwame Nkrumah University of Science and Technology, Kumasi, Ghana; 3 Department of Population, Family and Reproductive Health, School of Public Health, Kwame Nkrumah University of Science and Technology, Kumasi, Ghana

**Keywords:** Community-based health planning and services (CHPS), Access to healthcare, Healthcare delivery, Sefwi Wiawso, Ghana

## Abstract

**Background:**

In spite of the successes of the community-based health planning and services (CHPS) policy since its inception in the mid-1990s in Ghana, data pertaining to the implementation and use of CHPS facilities in Sefwi Wiawso Municipal is scant. We assessed access to healthcare delivery and factors influencing the use of CHPS in Sefwi Wiawso Municipal.

**Methods:**

An analytical community-based cross-sectional study was conducted in the Sefwi Wiawo Municipal from September to October 2020. Respondents for the study were recruited through multi-stage sampling. Information was collected on their socio-demographic characteristics, knowledge and use of CHPS facilities through interviews using a structured pre-tested questionnaire. Factors influencing the use of CHPS facilities were assessed using univariable and multivariable logistic regression to generate crude and adjusted odds ratios (ORs) with 95% confidence intervals (CIs). *P* ≤ 0.05 was considered statistically significant.

**Results:**

A total of 483 respondents were recruited for the study. The mean age of the respondents was 43.0 ± 16.3 years, and over 70% were females or married/cohabiting with their partners. Most respondents (88.2%) knew about the CHPS concept and more than half (53.4%) accessed healthcare in the CHPS facilities. Most respondents rated the quality of health services (> 65%) and staff attitude (77.2%) very positively. Significant factors influencing the use of the CHPS facilities were; knowledge of the CHPS concept (AOR 6.57, 95% CI 1.57–27.43; *p* = 0.01), longer waiting time for a vehicle to the facility, and shorter waiting time at the facility before being provided with care. People who waited for 30–60 min (AOR 2.76, 95% CI 1.08–7.07; *p* = 0.01) or over an hour (AOR 10.91, 95% CI 3.71–32.06; *p* = 0.01) before getting a vehicle to the facility, while patients who waited for less than 30 min (AOR 5.74, 95% CI 1.28–25.67; *p* = 0.03) or 30–60 min (AOR 2.60, 95% CI 0.57–11.78; *p* = 0.03) at the CHPS facility before receiving care were more likely to access care at the CHPS facilities.

**Conclusion:**

Knowledge, and use of healthcare services at the CHPS facilities were high in this population. Interventions aimed at reducing waiting time at the CHPS facilities could greatly increase use of healthcare services at these facilities.

## Introduction

Globally, healthcare systems still fall short of providing accessible, quality, comprehensive, and integrated care [1]. Major stakeholders, policy planners, development partners, and healthcare decision-makers require a better understanding of the Primary Health Care (PHC) concept. In 2005, the Ministry of Health and the Ghana Health Service adopted Community-based Health Planning and Services (CHPS) as a nationwide strategy for delivering primary health care services to address a myriad of health challenges confronting the majority of the people residing in rural areas[2, 4] Using the CHPS initiative together with other interventions, the Ghana Health Service has made several strides in achieving universal health coverage over the years [5].

The CHPS policy as a community-initiated health intervention is unique given its acclamation as a remarkable innovation to reducing inequalities in accessing health care delivery[6, 7]. It has advanced primary health care in Ghana by improving access to healthcare services, enhancing equity, and increasing coverage of essential healthcare services. It has specifically targeted underserved communities, addressing disparities in access and providing a wide range of services including maternal and child health, family planning, preventive health care such as immunizations, and treatment for common ailments[3,4, 8, 9]. CHPS has demonstrated tangible impacts on health outcomes, including reductions in maternal and child mortality rates and improvements in healthcare-seeking behaviors [10, 12] Moreover, it has proven to be cost-effective, efficient, scalable, and sustainable, making it a viable model for PHC in Ghana [3, 8].

Despite the successes of the CHPS policy, some barriers mitigating access to health care among the rural populace have been identified [12, 16]. Consequently, the CHPS concept is not meeting its expected outcomes due to several factors[12, 15]. These include lack of practical understanding of CHPS implementation by district-level managers; CHPS evolving into static clinic services focused on constructing health posts rather than its community-driven approach; managers often delaying CHPS implementation, waiting for central government resources instead of mobilizing local community resources; no central government budgetary allocation to cover startup costs as anticipated; heavy investment in CHPS staff recruitment and training without accompanied adequate investment in equipment; and poor leadership and supervision hindering effective implementation[14, 17].

Previous studies on CHPS concept in Ghana have focused on access to maternal health, family planning, and child health services[6, 9, 10, 18], with few studies looking at CHPS utilization among the general populace [19]. Anecdotal evidence suggests that the CHPS implementation processes have been inconsistent in Sefwi Wiawso Municipal, coupled with logistical and organizational challenges. There is limited information on the contribution of CHPS to health care access in Sefwi Wiawso Municipal. This study examined access to healthcare delivery as well as factors influencing the use of CHPS in the municipal.

## Methods

### Study design

This is an analytical cross-sectional study that was conducted in 21 selected communities within the Sefwi Wiawso Municipality from September to October 2020.

### Study setting

Sefwi Wiawso is the capital town of the Western-North Region of Ghana, which has a population of 151,220 with similar proportions of males (50.1%) and females (49.9%) [20]. The municipality has 135 communities. Majority of the communities are engaged in active agricultural activities in rural communities. Hence traveling to the few health facilities to access health care services is often associated with diverse challenges stemming from distance barriers, poor road network, irregular transport system as well as socio-cultural beliefs(20). These pose a great threat to rural communities in the Sefwi Wiawso Municipal especially in accessing health care at the CHPS facilities. The municipal is divided into seven sub-municipals to enhance comprehensive public health coverage. The sub-municipals are: Wiawso, Datano, Paboasi, Anyinabrim, Abrabra, Asafo and Asawinso. The municipal has 35 health facilities comprising four hospitals, two clinics, three health centres, one maternity home and 25 CHPS facilities. The CHPS facilities are Abrabra, Nkonya, Sui, Akurafo, Boako, Bechiwa, Aboagyekrom, Domeabra, Bowobra-Appentemedi, Datano, Ahukwa, Nyamegyiso, Nsuonsua, Aboduam, Bosomoiso, Aboanidua, Ahwiaa, Ahwiam, Ntrentrenso, Amafie, Futa, Akoti-Etwebo, Penakrom-Nyamebekyere, Old Adiembra, and Watico CHPS [21].

### Sample size determination

Sample size for the study was estimated using Epi Info, version 7.1.1.14 (Centers for Disease Control and Prevention, Atlanta, GA, USA) at 80% power, with 95% confidence level and 5% margin of error. It was assumed that the factors influencing utilization of the CHPS services in Sefwi Wiawso were similar to those observed in Kintampo by Wiru et al.[19], in terms of residents’ age, education and income status, and by allowing for 10% contingency, an estimated sample size of 483 was determined to have adequate power to detect the factors influencing use of CHPS facilities in Sefwi Wiawso Municipal.

### Selection of respondents

Individuals who were at least 18 years old, had been resident in the municipal for at least six months and had visited a health facility within the six months preceding the study were eligible for inclusion into the study. Individuals who were unwilling or unable to provide consent were excluded.

The respondents for the study were selected through multi-stage sampling. The sub-municipals and communities were selected by simple random sampling through balloting using the lists of sub-municipals and communities respectively as the sampling frames. Seven sub-municipals with at least three communities each were chosen for the study. The number of individuals selected from each sub-municipal was in proportion to the size of the sub-municipal. The estimated number of household members in each sub-municipal was obtained from the Municipal Health Directorate.

In each community, a central location such as the chief’s palace, a church or mosque was chosen. A pen was spun and the first house in the direction the pen pointed to was selected. Walking in the chosen direction, every other house was selected. If a selected house was locked, the research assistants made multiple attempts to contact occupants of the house. If the occupants could not be reached or were not available, they moved to the next available house. Within each selected household, one eligible household member was chosen by balloting and invited to participate in the study. Where there was only one eligible person, the individual was invited to participate. Selected households that had no eligible respondents were replaced by the next consecutive household. In the event that the expected number of respondents for that community was not achieved, the researcher returned to the reference point and the procedure was repeated until the desired number was attained.

After explaining the purposes and benefits of the study, each consenting individual was invited to participate in the study. The respondents were assured of confidentiality of the information that was collected during the study. Consenting individuals were interviewed in English, Twi or Sefwi (vernacular) using a pre-tested structured questionnaire comprising closed and open-ended questions. The respondents were interviewed face-to-face with an android tablet loaded with the questionnaire and enabled with Open Data Kit (ODK). Data was collected on their socio-demographic characteristics, awareness and access to service delivery, and factors influencing their access to health service delivery. During the interview process amid the Coronavirus Disease 2019 (COVID-19) pandemic, safety measures were rigorously implemented to safeguard all individuals involved in the research activities. All respondents and research assistants washed their hands with soap and water, or used hand sanitizers, and were provided with face masks if needed. They were educated on proper face mask usage and maintained a physical distance of at least 1 m throughout the interview. Additionally, they also complied with other COVID-19 restrictions imposed by national or local authorities.

The questionnaire was translated into Twi and Sefwi, and back translated into English. Pre-testing was done among 20 respondents who were conveniently selected in two communities in Juaboso District, ensuring representation of the target population’s demographic characteristics and language proficiency. Participants provided feedback on the clarity, language, and overall comprehensibility of the questionnaire, guiding revisions to improve reliability and validity. Overall, all participants indicated the language was easy to understand, and most found the questions easy to understand and answer. Revisions included clarifying ambiguous questions, and ensuring logical flow. The revised schedule underwent further review by the research team and experts to ensure its effectiveness for the main study. We employed cognitive interviewing and expert review to enhance the reliability and validity of the questionnaire.

### Study variables

The main outcome variable for the study was use of CHPS facility. This was measured as respondents’ usage of CHPS facility for healthcare services during the year prior to the study. Those who visited CHPS were coded as 1 (yes) and those who used other health facilities such as the district hospital, private hospitals/clinics, faith-based, or any other form of health facility were coded as 0 (no).

The independent variables were age, gender, marital status, educational level, occupation, monthly income, household size, cost of transportation, time spent before getting access to vehicle to CHPS facility (measured in minutes), waiting time at the CHPS facility, satisfaction with cost of services at CHPS facilities, satisfaction with availability of drugs and suppliers and basic equipment, knowledge of the CHPS concept, and staff attitude. These variables were chosen on the basis of biologic plausibility and evidence from literature [3, 7, 14, 22].

### Data analysis

Data were cleaned and analyzed using Stata version 15 (Stata Corp, College Station, Texas, USA). Descriptive statistics were used to summarize clients’ perception of the quality of health care provided at the CHPS facilities. Categorical variables were compared using the chi-square [χ^2^]. Factors associated with use of CHPS facilities were examined using univariable and multivariable logistic regression to estimate crude and adjusted odds ratios (ORs) with 95% CIs. Univariable analysis were performed to examine the association of each explanatory variable with the use of CHPS facilities and variables whose association reached statistical significance at *p*≤0.05 were included in a multivariable model. Significant explanatory variables were added one at a time and those which remained independently associated with use of CHPS facilities at *p*≤0.05 were retained until all variables in the model were significant at *p*≤0.05. Excluded explanatory variables were retested in the final model one at a time to confirm lack of association. Variance inflation factor was computed to test for multicollinearity. All missing values were excluded from the analysis.

## Results

A total of 483 study respondents were recruited into the study. The socio-economic and demographic characteristics of the study respondents are shown in Table 1. The mean age of the study respondents was 43.0 ± 16.3 years, range 18–92 years. Over half (52.8%) of the respondents were 40 years or older. Over 70% were females and a similar percentage were married/cohabiting. Majority (77.8%) were Akans. About 30% had no formal education and almost half (49.1%) had completed basic education (i.e. primary or junior high school). Almost three-quarters (74.5%) were in employment with nearly 20% earning over 500 Ghana cedis per month (equivalent to $87.72 at the time). The median household size was 6 (interquartile range = 4–8). Almost all (95.4%) of the respondents were permanent residents of the communities that they were interviewed.

**Table 1:** Socio-economic and demographic characteristics of study respondents

Variable	Frequency (*N* = 483)	Percentage (%)
Age group (in years)		
< 30	113	23.4
30–39	115	23.8
40–49	102	21.1
50+	153	31.7
**Gender**		
Female	352	72.9
Male	131	27.1
**Marital Status**		
Single	67	13.9
Married/cohabiting	342	70.8
Separated/divorced/widowed	74	15.3
**Religion**		
Christianity	427	88.4
Islam Traditional religion	27 29	5.6 6.0
**Ethnicity**		
Akan	376	77.8
Mole-Dagbane	56	11.6
Ewe/Ga-Adangbe/Nzema	51	10.6
**Level of education**		
No formal education	147	30.4
Basic education	237	49.1
Secondary/tertiary	99	20.4
**Occupation**		
Unemployed	123	25.5
Semi-skilled	327	67.7
Skilled	33	6.8
**Monthly Income (Ghana cedis)**		
< 100	18	5.0
100–499	233	64.7
500–999	88	24.5
1000+	21	5.8
**Household size**		
1–5	209	43.3
6–10	227	47.0
11+	47	9.7
Median (interquartile range)	6 [4–8]	
**Residential status**		
Permanent	461	95.4
Temporal	22	4.6

### Community members’ knowledge of services provided at the CHPS facilities

From Table 2, most respondents (88.2%) had knowledge on the CHPS concept, with the commonest (56.6%) source of information being the community information centres. Less than a fifth (16.1%) of respondents obtained their information on CHPS through the community health workers. Majority (91.1%) knew the location of the CHPS facilities in their community and (81.4%) of the respondents were aware that the CHPS facilities provide curative services.

**Table 2:** Knowledge of respondents’ about chps services in the communities

Variables	Frequency (*N* = 483)	Percent (%)
**Respondents’ knowledge of the presence of CHPS facility**		
No	57	11.8
Yes	426	88.2
Source of knowledge on the CHPS facility*		
Media	145	34.0
Community durbar	23	5.4
Community Health Worker	71	16.7
Community Information Centre	241	56.6
Friends/family	215	50.5
**Knowledge on the location of a CHPS facility**		
No	43	8.9
Yes	440	91.1

*Multiple responses allowed

### Access to, use and services provided in CHPS facilities

From Table 3, about half (49.9%) of the respondents had a CHPS facility located in the communities that they resided in. More than half (53.4%) had visited the CHPS facility in their community to access healthcare during the past year with nearly two-thirds (65.5%) visiting the CHPS facility 2–5 times in the one year preceding the study. Common reasons for accessing care at the CHPS facility included treatment for minor ailments (65.9%) and proximity to their residence (47.7%).

Multiple means of transportation were used by respondents when accessing healthcare services from the CHPS facilities. About half of the respondents either walked (49.0%) or used public transport (51.4%) whereas 9.9% indicated that they used motorbikes or bicycles to the CHPS facility to access healthcare. Among those who used public transport to the CHPS facility, almost two- thirds (66.2%) indicated that they spent at least GHS 6.00 (a little over $1 at the time of the survey) on transportation to and from the health facility. Waiting time to access vehicle to the health facility for most of the respondents (84.6%) was up to an hour (see Table 3).

About 86% of the respondents had registered with the National Health Insurance Scheme (NHIS), with only 58% having valid NHIS cards as at the time of the survey and 54% obtained their prescribed medications from the CHPS facility. Most respondents considered the staff at the CHPS facilities to be friendly (87.6%) and competent (76.7%). Altogether, over three-quarters (77.2%) of the respondents considered the staff attitude towards them in the facility to be good/very good (see Table 3).

**Table 3:** Access to, use and services provided in CHPS Facilities in the Sefwi Wiawso Municipal, Ghana

Variable	Frequency (*N* = 483)	Percent (%)
**Type of health facility in your community**		
None	210	43.5
CHPS	241	49.9
District hospital	13	2.7
Faith-Based hospital	3	0.6
Private hospital	4	0.8
Health centre	4	0.8
Private clinic	8	1.7
**Usage of CHPS facility during the past year**		
No	225	46.6
Yes	258	53.4
Frequency of CHPS facility access for care in the past year (*n* = 258)		
Once	62	24.0
2–5	169	65.5
6 or more times	27	10.5
Peculiar reasons for accessing healthcare at the CHPS facility (*n* = 258)*		
Minor ailments	170	65.9
Proximity to residence	123	47.7
Services are good	27	10.5
Services were less costly	77	29.8
Others	5	1.9
Means of transport to the nearest CHPS facility*		
Foot	243	49.0
Vehicle Motor bike/tricycle/bicycle	253 38	51.4 9.9
Transport fares incurred in accessing services (Ghana Cedi) [*n* = 260]**		
< 5	88	33.9
6–10	123	47.3
> 10	49	18.8
Time spent before getting access to vehicle to CHPS facility (minutes) [*n* = 208]		
< 30	77	37.0
30–60	99	47.6
> 60	32	15.4
Current registration status on NHIS		
No	66	13.7
Yes	417	86.3
**Validity status of respondents’ NHIS cards**		
No	201	41.6
Yes	282	58.4
Respondents’ assessment of the quality of service delivery*		
Friendly attendants	422	87.6
Competent staff	369	76.6
Less waiting time	315	65.4
Relevance of services to respondent’s need	174	36.1
Well-equipped facility	57	11.8
**Respondent’s ability to purchase medicines at the facility**		
No	223	46.2
Yes	260	53.8
**Average waiting time at the facility (from arrival till treatment is complete(minutes)**		
< 30	256	53.0
30–60	195	40.4
> 60	32	6.6
**Respondents’ perception of staff attitude**		
Bad/very bad	14	2.9
Moderate	96	19.9
Good/very good	373	77.2

* Multiple responses allowed, Exchange rate $1 = 5.70 Ghana cedis, **223 respondents walked or used bicycle

### Factors influencing use of CHPS facilities

Factors influencing the use of the CHPS facilities are shown in Table 4. On univariable analysis, age group, marital status, time spent before getting access to a vehicle to the CHPS facility, waiting time at the CHPS facility, satisfaction with cost of services, knowledge of the CHPS concept and respondents’ perception of staff attitude were significantly associated with the use of the CHPS facilities. On multivariable analysis, the time spent before getting access to a vehicle to CHPS facility, waiting time at the CHPS facility and knowledge of the CHPS concept remained significantly associated with CHPS facility utilization. The odds of using a CHPS facility increased with duration of waiting for a vehicle to a facility. Waiting for 30–60 min and over one hour before getting a vehicle to a facility increased the odds of using a CHPS facility by more than two and half (AOR 2.76, 95% CI 1.08–7.07) and nearly 11 times (AOR 10.91, 95% CI 3.71–32.06) respectively compared to patients who waited for less than 30 min for a vehicle. The likelihood of using a CHPS facility decreased with waiting time at the facility. Patients who waited for less than 30 min were over five and a half times more likely (AOR 5.74, 95% CI 1.28–25.67) and those who waited for 30–60 min were more than two and a half times likely (AOR 2.60, 95% CI 0.57–11.78) to use CHPS facilities compared to their counterparts who waited for over an hour. Having knowledge of the CHPS concept increased the odds of using CHPS facility by more than six and a half times (AOR 6.57, 95% CI 1.57–27.43) compared to patients who had no knowledge of the concept.

**Table 4:** Factors influencing use of CHPS facilities among respondents

Variable	Use of CHPS *n* = 258 (%)	COR (95%CI)	*P*-value	AOR (95%CI)	*P*-value
**Age group (years)**			0.05		0.12
< 30	50 (19.4)	1		1	
30–39	70 (27.1)	1.96 (1.16–3.32)		2.37 (0.76–7.35)	
40–49	58 (22.5)	1.67 (0.97–2.84)		3.67 (1.17– 11.49)	
50+	80 (31.0)	1.38 (0.85–2.25)		1.54 (0.51–4.63)	
**Gender**			0.84		
Female	189 (73.3)	1			
Male	69 (26.7)	0.96 (0.64–1.43)			
**Marital Status**			0.02		
Single	26 (10.1)	1		1	0.95
Married/cohabiting	190 (73.6)	2.03 (1.19–3.47)		1.21 (0.38–3.82)	
Separated/divorced	42 (16.3)	2.12 (1.08–4.15)		1.17(0.27–4.99)	
**Education**			0.21		
No formal education	79 (30.6)	1			
Primary/JHS	135 (52.3)	1.14 (0.75–1.72)			
SHS/Vocational	35 (13.6)	0.70 (0.40–1.22)			
Tertiary	9 (3.5)	0.64 (0.26–1.62)			
**Occupation**			0.26		
Skilled	16 (6.2)	1			
Semi-skilled	183 (70.9)	1.35 (0.66–2.76)			
Unemployed	59 (22.9)	0.98 (0.45–2.11)			
**Monthly income (GHC)**			0.06		
< 100	5 (2.5)	1			
100–499	136 (68.4)	3.64 (1.26–10.56)			
500–999	48 (24.1)	3.12 (1.02–9.50)			
1000+	10 (5.0)	2.36 (0.62–9.03)			
**Household size**			0.18		
< 5	102 (39.5)	1			
6–10	131 (50.8)	1.43 (0.98–2.90)			
> 10	25 (9.7)	1.19 (0.63–2.25)			
**Cost of transportation to health facility (GHC)**			0.06		
< 5	32 (44.4)	1			
5–10	30 (41.7)	0.56 (0.31–1.03)			
> 10	10 (13.9)	0.49 (0.20–1.02)			
**Time spent before getting access to vehicle to CHPS facility (minutes)**			0.05		0.01
< 30	10 (19.2)	1		1	
30–60	23 (44.2)	2.03 (0.90–4.57)		2.76 (1.08–7.07)	
> 60	19 (36.6)	9.79 (3.71–25.81)		10.91 (3.71–32.06)	
**Waiting time at the CHPS facility (minutes)**			0.01		0.03
< 30	174 (67.4)	7.58 (3.15–18.24)		5.74 (1.28–25.67)	
30–60	77 (29.9)	2.33 (0.96–5.65)		2.60 (0.57–11.78)	
> 60	7 (2.7)	1		1	
**Satisfaction with affordability of CHPS services**			0.01		0.14
Not satisfied	33 (12.8)	1		1	
Satisfied	197 (76.4)	2.44 (1.51–3.92)		2.06 (0.84–5.07)	
Very Satisfied	28 (10.8)	2.82 (1.36–5.86)		3.52 (0.92– 13.49)	
**Satisfaction with availability of drugs and suppliers and basic equipment**			0.09		
Not satisfied	13 (5.1)	1			
Satisfied	239 (92.6)	1.79 (0.86–3.72)			
Very Satisfied	6 (2.3)	0.80 (0.23–2.70)			
**Knowledge of the CHPS concept**			0.01		0.01
No	16 (6.2)	1		1	
Yes	242 (93.8)	3.37 (1.83–6.19)		6.57 (1.57–27.43)	
**Respondents’ perception of staff attitude**			0.01		0.53
Good	218 (84.5)	1.87 (0.64–5.51)		3.08 (0.38–33.48)	
Moderate	34 (13.2)	0.73 (0.23–2.28)		1.07 (0.45–2.51)	
Bad	6 (2.3)	1		1	

AOR-Adjusted odds ratio: CHPS-Community-based Health Planning and Services; CI-Confidence interval; COR- Crude odds ratio; GHC-Ghana cedis; JHS-Junior High School; SHS-Senior High School

## Discussion

This study assessed factors influencing the use of CHPS facilities in a predominantly rural district in Ghana. Majority of the respondents knew about the CHPS concept as well as the services being provided. Most respondents accessed health care from the CHPS facility in their community at least twice in the year. The waiting time for majority of the respondents was up to one hour and they rated the competence and performance of the healthcare providers very positively. Significant determinants of the utilization of CHPS facilities were time spent before getting a vehicle to the CHPS facility, waiting time at the CHPS facility and knowledge of the CHPS concept.

The finding that more than half (53.4%) of the respondents had visited the CHPS facilities to access healthcare during the past year highlights the significant utilization of CHPS facilities among the study population. This finding is consistent with those of previous studies conducted in Kintampo North Municipality of the Bono East Region and Komenda-Edina-Eguafo-Abrem Municipality of the Central Region of Ghana, where high proportions of community members utilized the services of CHPS services for healthcare services [19, 23]. This high utilization rate underscores the importance and relevance of CHPS facilities in providing essential healthcare services to the community. Johnson et al. argued that access to a CHPS facility is associated with utilization of healthcare services within the facility, which increases significantly with proximity to the CHPS facility [10]. Similarly, we observed that most of the respondents accessed health care services from the CHPS facility at least twice a year, where over 47% cited proximity to the CHPS facility as one of the main considerations.

Contrary to our expectation, respondents who experienced longer waiting time before accessing transportation to the facility were more likely to utilize CHPS. This could possibly be due to limited alternative options or a perceived urgency in seeking healthcare for individuals who experienced longer waiting times before accessing transportation to the facility. Given the predominantly rural nature of the communities in the municipal, healthcare services in most of these communities are largely provided by CHPS facilities and a few or no hospitals [20, 21, 24]. This could mean that despite the longer waiting time for transportation, there might be limited alternative options. It is also conceivable that respondents waiting for over an hour before getting access to CHPS facilities do so on the premise of the perceived quality of services they anticipate to obtain, the extent to which their health needs are met at the facility and hence not deterred by the long waiting time for vehicle to the CHPS facility to seek healthcare services. This is supported by the findings of Assan et al. where users of CHPS facilities were highly satisfied with the services provided at the CHPS and the positive attitude of community health professionals [17]. The long waiting time for transportation to CHPS facilities also highlights some of the challenges of accessing health care in such rural communities. Transportation to healthcare facilities in rural areas are problematic and hinders access to care in most communities in Ghana [25]. Therefore, improving geographic access to CHPS facilities is essential to universal health coverage [26].

Our finding that shorter waiting times at the facility were associated with increased odds of using of CHPS facilities is consistent with those of previous studies which identified long waiting time as a significant challenge to seeking healthcare services[5, 13, 17, 22]. Arguably, patients who spend less time at the CHPS facility may be more inclined to seek care, reflecting a positive patient experience and potentially higher levels of satisfaction with the healthcare services provided [5, 13, 17, 22]. The implementation of COVID-19 preventive measures, such as physical distancing requirements, wearing of face masks, and hand hygiene practices, likely influenced transportation facilities’ utilization and waiting times at the CHPS facility. These measures may have led to changes in travel behavior, increased waiting times due to screening protocols or reduced facility capacity, and altered patient-provider interactions.

Respondents’ knowledge of the CHPS concept increased their likelihood of using CHPS, underscoring the importance of patient understanding of the CHPS concept. Individuals who are better informed of the CHPS concept may be more inclined to use CHPS facilities, recognizing the benefits of community-based healthcare delivery and the availability of essential health services [15]. The majority of our study respondents knew about the CHPS concept as well as the services being provided. Their commonest source of information was the community information centre. These information centres are major sources of information in rural communities and most inhabitants listen to them, explaining the high level of knowledge observed in the study setting. Interestingly, less than a fifth of the respondents got their information on CHPS through the community health workers, indicating that the Municipal Health Directorate needs to intensify campaign efforts provided by the community health workers. Johnson et al.[10] revealed that health education sessions within CHPS facilities should prioritize addressing prevailing health problems, preventive measures, and care practices.

### Strengths and weakness of the study

This study contributes to the literature by exploring factors influencing access to a broad scope of health care services within the CHPS system in largely rural communities. We uniquely investigated access to a wider scope of healthcare services within rural communities where the CHPS concept is predominantly operational. Key strengths of our study include, being a population-based study involving geographically diverse communities where respondents were randomly selected, the findings are generally representative of the diverse ethnic groups in the municipal and similar communities. However, the study has some limitations. First, only persons who were at least 18 years old, had visited a facility within the six months preceding the study and were available at home during the period were recruited into the study. The experiences of the younger ones (< 18 years) and those who were not at home during the research could be different and would have been worthwhile. Second, soliciting the views of the health care providers and municipal health directorate staff would have been useful especially their challenges in implementing the policy.

## Conclusion

Knowledge of the CHPS concept and the use of healthcare services at CHPS facilities were high in this predominantly rural population. Maintaining awareness campaign strategies on the CHPS concept such as use of the community information centres and intensifying community health worker campaign efforts would be worthwhile. Interventions aimed at reducing the waiting time at the CHPS facilities could significantly improve inhabitants’ use of healthcare services in these facilities.

## Data Availability

The data used for this study are available upon reasonable request from the corresponding author.

## Abbreviations

CHPS: Community-based Health Planning and Services
CHRPE: Committee on Human Research Publication and Ethics
CI: Confidence Interval
GHS: Ghana Health Service
GSS: Ghana Statistical Service
JHS: Junior High School
NHIS: National Health Insurance Scheme
ODK: Open Data Kit
OR: Odds Ratio
PHC: Primary Health Care
SHS: Senior High School
WHO: World Health Organization
